# Radioresistant mouse pheochromocytoma cell lines

**DOI:** 10.3389/fonc.2025.1517132

**Published:** 2025-07-30

**Authors:** Sandy Lemm, Marcel Gebhardt, Thomas Groß, Susan Richter, Martin Ullrich, Jens Pietzsch

**Affiliations:** ^1^ Institute of Radiopharmaceutical Cancer Research, Department of Radiopharmaceutical and Chemical Biology, Helmholtz-Zentrum Dresden-Rossendorf, Dresden, Germany; ^2^ Faculty of Chemistry and Food Chemistry, School of Science, Technische Universität Dresden, Dresden, Germany; ^3^ Institute for Clinical Chemistry and Laboratory Medicine, University Hospital Carl Gustav Carus, Medical Faculty Carl Gustav Carus, Technische Universität Dresden, Dresden, Germany; ^4^ Core Unit for Molecular Tumor Diagnostics, National Center for Tumor Diseases/University Cancer Center Dresden, University Hospital Carl Gustav Carus, Medical Faculty Carl Gustav Carus, Technische Universität Dresden, Dresden, Germany; ^5^ Auckland Cancer Society Research Centre, School of Medical Sciences, University of Auckland, Auckland, New Zealand

**Keywords:** radioresistance, X-ray, pheochromocytoma, paraganglioma, irradiation, HIF-2α, pseudohypoxia

## Abstract

**Objective:**

Patients diagnosed with metastatic pheochromocytoma/paraganglioma (PCC/PGL) have limited treatment options. In some cases, peptide receptor radionuclide therapy (PRRT) is followed by an eruption of metastases, possibly originating from tumor cells with a radioresistant phenotype. However, the underlying mechanisms of radioresistance in PCC/PGL remain largely unknown and appropriate models are missing.

**Methods:**

Two genetically modified mouse pheochromocytoma (MPC) cell lines, one positive and one negative for hypoxia-inducible factor 2α expression (MPC+HIF-2α and MPC+EV [empty vector], respectively), were X-ray-conditioned through fractionated irradiation at sublethal doses. Two procedures were tested: one allowed for recovery between each irradiation step (recIR), while the other demanded daily irradiation (dayIR). Changes in cell morphology, growth rates, and DNA repair (γH2AX immunostaining) were characterized in response to irradiation.

**Results:**

We generated two MPC+HIF-2α- and two MPC+EV-derived cell lines that tolerate irradiations with X-rays at dose fractions of 2 Gy per day without significant growth inhibition. All recIR-and dayIR-conditioned cell lines showed increased DNA repair capacity. Morphological changes toward stronger clustering and slower growth were more pronounced in dayIR-conditioned than in recIR-conditioned cell lines. X-ray-conditioned MPC+HIF-2α cells showed the highest increase in resistance to X-ray-treatment with dose fractions up to 5 Gy per day.

**Conclusion:**

The herein established X-ray-conditioned MPC cell lines represent PCC/PGL models with a radioresistant phenotype. Further investigations on the radiation-induced genetic responses of these cell lines, their corresponding tumor spheroids, and tumor allografts in mice will help to elucidate the underlying mechanisms of acquired radioresistance and radionuclide therapy-induced metastatic eruption in PCC/PGL. Lastly, the suitability of advanced PRRT and complementary treatments can be tested to improve theranostic strategies.

## 1 Introduction

Pheochromocytomas and paragangliomas (PCC/PGL) are mostly benign, catecholamine-producing, neuroendocrine tumors arising from chromaffin tissue of the adrenal medulla or extra-adrenal sympathetic or parasympathetic ganglia. They are broadly categorized into three genetic clusters: Cluster 1 (pseudohypoxia) includes mutations in succinate dehydrogenase subunit, *VHL*, and *EPAS1* genes, leading to disrupted cellular oxygen sensing. Cluster 2 (kinase signaling) involves mutations in *RET*, *NF1*, and *TMEM127* genes, which activate kinase signaling pathways. Cluster 3 (Wnt signaling) is less common and involves *CSDE1* and *MAML3* fusions, associated with Wnt signaling alterations (1, 2). Owing to the tumor-derived hormone production (e.g. adrenalin or noradrenalin), patients suffer from various symptoms, including hypertension, palpitations, headaches, sweating and anxiety. Regardless of their rare occurrence of 2−8 cases per million (3–5), PCC/PGL remain of clinical importance, since they all have metastatic potential (6) and approximately 10% are at risk of tumor relapse after surgical resection (7). Meta-[^131^I]iodobenzylguanidine ([¹³¹I]MIBG) is one well-known treatment option, while peptide receptor radionuclide therapy (PRRT) targeting the somatostatin type 2 receptor (SSTR2) with [^177^Lu]DOTA-(Tyr^3^)octreotate ([^177^Lu]DOTA-TATE) is increasingly being considered for slow growing lesions. However, metastatic outbursts after initially successful PRRT have been reported in some cases months or years after PRRT (8, 9). Based on the physical half-life of radionuclides used for PRRT (e.g. 6.7 days for lutetium-177) the initially high dose inhibits (or “controls”) most of the PCC/PGL cells, but those that can escape the high-dose phase may acquire radioresistance during the following low-dose radiation phase.

Several mechanisms facilitate radioresistance, such as genetic or epigenetic remodeling leading to adjusted DNA damage repair, modulated microenvironment and regulation in several signaling pathways. Especially, the role of hypoxia inducible factors (HIF) and their related signaling pathways are of importance since hypoxia (low oxygen levels) is a common feature of the tumor microenvironment, that also associates with downstream transcriptional changes that can activate oncogenic pathways (10–12). Gene expression analysis together with immunohistochemical studies have shown induction of HIF-2α, but not HIF-1α, specifically in cluster 1 PCC/PGL (13–16).

We hypothesize that PCC/PGL cells irradiated with sublethal doses of ionizing radiation (X-ray-conditioning) acquire a radioresistant phenotype characterized by increased DNA repair capabilities enabling sustained proliferation compared to cultivation-matched controls. An increased DNA repair machinery after X-ray-treatment has already been reported for other cell types (17). It is further assumed that proliferation rates and survival of PPC/PGL cells are higher after X-ray-conditioning (18, 19). Based on previously published links between HIF-2α and radioresistance (20, 21), we expect that HIF-2α modulates the molecular and proliferative responses of radioresistant PCC/PGL cells.

To investigate radioresistance in PCC/PGL, mouse pheochromocytoma (MPC) cells were conditioned under periodic X-ray irradiations to adapt their phenotype to sublethal dose fractions. X-ray-conditioning was performed using two different irradiation regimes previously reported by Kuwahara et al. and Fukuda et al. (18, 22). Following these X-ray-conditioning protocols, the potential acquisition of a radioresistance phenotype was evaluated by independent X-ray-treatment regimes.

In this study, we compare the applied irradiation protocols for X-ray-conditioning regarding their applicability and the resulting cellular morphological responses at sublethal radiation doses. Furthermore, we show the differences in radioresistant cells compared to their cultivation-matched counterparts in terms of proliferation, HIF-2α levels, DNA damage repair, and survival in response to further irradiation at higher doses.

## 2 Materials and methods

### 2.1 Cell culture

All cell lines utilized in this work originated from mouse pheochromocytoma cells [MPC, clone 4/30PRR (23)] with additional genetic modifications. Specifically, two genetically modified mCherry-positive MPC cell sublines differing in their capability for HIF-2α signaling were included in the present study: MPC^mCherry^+HIF-2α cells expressing a codon-optimized *EPAS1* gene [“*HIF-2α* transgene” encoding for HIF-2α, with confirmed expression on mRNA and protein level (24)] and MPC^mCherry^+EV control cells harboring an ‘empty vector’ (without the *EPAS1* expression cassette) (25). Given their common mCherry-positive phenotype, the cells are referred to as MPC+HIF-2α and MPC+EV throughout the publication. Cells were maintained in RPMI with 10% (v/v) horse serum, 5% (v/v) fetal bovine serum, and 250 ng/mL G418 (Gibco, Paisley, UK) to select for genetically modified clones. During experiments, the antibiotic was replaced by 1% (v/v) penicillin/streptomycin (Gibco, 10.000 U/mL), because of formerly detected radiosensitizing effects of G418 (20). Cells were detached using trypsin and subcultivated as monolayers on collagen-coated flasks or microplates. Unless stated otherwise, cells were routinely incubated at 37°C in humidified normoxic atmosphere with 5% (v/v) CO_2_.

### 2.2 Cell irradiation with X-rays

Cells grown in monolayer were irradiated with a 200 kV photon beam using a Maxishot X-ray system equipped with a Y.TU/320-D03 tube (YXLON, Hamburg, Germany). Prior to each irradiation session, the dose rate delivered by the system was confirmed to be in the range of 1.154 – 1.213 Gy/min.

### 2.3 X-ray-conditioning of cells

To generate MPC cell lines with a radioresistant phenotype, two different protocols for X-ray-conditioning at fractionated sublethal irradiation doses ≤ 2 Gy were applied (**Table 1**). The first protocol was published by Fukuda et al. in 2004 (22) allowing cells to recover between each irradiation step (recIR): Cells at 50% confluence were irradiated with dose fractions of 2 Gy followed by recovery between two to four days until monolayers reached 80% confluence. Cells were subcultivated and irradiated again at 50% confluence. This cycle was repeated until a cumulated dose of 60 Gy was absorbed. The second protocol was based on daily irradiations with stepwise increasing X-ray doses published by Kuwahara et al. in 2017 (18), with some modifications (dayIR): Cells at mid to high confluency were irradiated starting with dose fractions of 0.5 Gy/d for 5 days, followed by 1.0 Gy/d for 10 days, 1.5 Gy/d for at least 15 days and, if cells continued proliferating, final irradiation with 2.0 Gy/d for 30 days. Irradiation was suspended on weekends or when cells stopped proliferating. When cells continued to grow, irradiation was continued starting again with dose fractions of 1.5 Gy per irradiation. This procedure was repeated until cells continued to proliferate under daily irradiation with dose of 2 Gy/day.

**Table 1:** Overview of reference work for X-ray-conditioning.

Reference	Kuwahara et al., 2017	Fukuda et al., 2004
Underlying irradiation process	Daily irradiation with increasing doses from 0.5 Gy to 2 Gy per day	At 50% confluency irradiation with 2 Gy
Minimum irradiation cycles required	5 × 0.5 Gy	30 × 2.0 Gy
	10 × 1.0 Gy	
	15 × 1.5 Gy	
	30 × 2.0 Gy	
	**∑ 60 cycles**	**∑ 30 cycles**
Total doses cumulated at endpoint	95 Gy (assuming continued cell proliferation)	60 Gy
Stable phenotype without maintenance irradiation	1 month	3 months

Bold values indicate minimal irradiation cycles need to reach X-ray-conditioned cell lines according to reference protocol.

Cells were cultured as monolayers and recovered from the previous irradiation for at least four hours before subcultivation. Prior to X-ray-treatment experiments, cells recovered for two weeks after the last X-ray-conditioning irradiation. To maintain radiation-induced phenotypic adaptations, X-ray-conditioning was resumed after a recovery of four weeks. The original MPC+HIF-2α and MPC+EV cells were maintained in routine culture without any X-ray-conditioning and served as cultivation-matched controls (noIR).

### 2.4 Monitoring of cell growth *in vitro* after X-ray-conditioning

Cells (1×10^4^/well) were seeded in collagen-coated flat bottom 96-well plates, followed by 15 min settlement at room temperature. Plates were transferred to the IncuCyte S3 live-cell analysis system (Sartorius, Göttingen, Germany) and incubated at routine conditions. Growth was monitored with an interval of 6−8 hours until maximal confluence was reached. Media was changed every 2−3 days with 50% exchange. Cell growth in each well was analyzed with IncuCyte 2019B Rev2 software (Essen BioScience Inc., Essen, Germany). Growth rates (*k*) were determined from nonlinear regression analysis using logistic growth fitting as implemented in Prism 10 (GraphPad Software, Boston, MA, USA).

### 2.5 Detection of *HIF-2α* transgene expression after X-ray-conditioning

#### 2.5.1 RNA isolation

At indicated time points, cells were harvested with trypsin and pellets were snap-frozen in liquid nitrogen. RNA was isolated utilizing the miRNeasy Tissue/Cells Advanced Kit (Qiagen, Beverly, MA, USA).

#### 2.5.2 Quantitative reverse transcriptase polymerase chain reaction

Gene expression was measured via qRT-PCR using the CFX Connect Real-Time PCR Detection System (Bio-Rad, Hercules, CA, USA) as described previously (25). For each sample, 1 µg of total RNA was amplified by double-strand cDNA synthesis (qScript^®^ cDNA Synthesis Kit, Qiagen). Gene expression analysis was performed by qPCR with the “PerfeCTa^®^ SYBR^®^ Green SuperMix” (Avantor, Radnor, PA, USA) and measured using the CFX384 Real-Time PCR Detection System (Bio-Rad). The following primers were used: Actin forward 5’- GGCTGTATTCCCCTCCATCG -3’ and reverse 5’- CCAGTTGGTAACAATGCCATGT -3’; *HIF-2α* forward 5’-TCGACTCCTCTGACGATGTG-3’ and reverse 5’-CAGAGGGCTCGTCAAAGTTC-3’; *neoR* forward 5’-AGACAATCGGCTGCTCTGAT3’ and reverse 5’-CTCGTCCTGCAGTTCATTCA-3’.

#### 2.5.3 Library preparation for RNA sequencing and data analysis

RNA libraries were prepared using the Illumina stranded mRNA Prep, Ligation Kit (Illumina, San Diego, CA, USA) according to the manufacturer’s instructions. Briefly, mRNA was purified from 500 ng of total RNA using oligo(dT) beads. Poly(A)+ RNA was fragmented to approximately 150 bp and converted into cDNA. The cDNA fragments were end-repaired, adenylated at the 3’ end, adapter-ligated, and amplified by 12 cycles of PCR. Library quantity was assessed using the dsDNA Broad Range Kit with the Qubit 2.0 Fluorometer (both Life Technologies, Carlsbad, CA, USA) and quality was evaluated using the NGS Fragment Kit (1−6000 bp) on the Fragment Analyzer 5200 (both Agilent Technologies, Santa Clara, CA, USA). All barcoded libraries were pooled and sequenced 2×75 bp paired-end mode on an Illumina NextSeq550, targeting a minimum of 10 million reads per sample.

Quality control of raw reads was performed using FastQC v0.12.1 (26), followed by the removal of low-quality sequences and adapter sequences using Trimmomatic v0.39 (27). Trimmed reads were aligned to the *Mus musculus* reference genome assembly GRCm39 (GCF_000001635.27_GRCm39) from the Genome Reference Consortium using STAR v2.7.11b (28). The genome reference index was generated by SAMtools v1.9 (29). To enable quantification of the codon-optimized *EPAS1* transcript, the corresponding sequence was added to the genome index prior to alignment. Feature counts were generated using FeatureCounts from Rsubread v2.18.0 (30), and differential expression analysis was conducted using the DESeq2 R package v1.20.0 (31). Transcripts per million mapped reads (TPM) values were calculated by normalizing raw read counts to gene length and total library size, enabling accurate comparison of gene expression levels across samples. MultiQC v1.14.0 (32) was utilized to generate the final quality control report including metrics from previous steps.

#### 2.5.4 Immunoblotting

Presence of HIF-2α in cell lysates was performed as described previously (24) with some modifications. In brief, cells were treated with 200 µM CoCl_2_ for 24 hours to stabilize HIF-2α. Cells were detached with 2 mmol/L EDTA and a cell scraper. Lysates were heated to 99°C for 1 min and transferred to a pre-casted 4-15% TGX Gel (BioRad, Hercules, CA, USA). Proteins were transferred to a PVDF membrane. Non-specific binding was blocked with 2% (w/v) bovine serum albumin and 5% (w/v) nonfat dried milk in Tris-buffered saline with 0.05% (v/v) Tween-20. HIF-2α was detected with the primary antibody NB100-122 (Novus Biologicals, Centennial, CO, USA; 1:500). The reference proteins β-actin (ACTB) and glycerinaldehyde phosphate dehydrogenase (GAPDH) were detected with the primary antibodies A0545 (Sigma-Aldrich, St. Louis, MO, USA; 1:5000) and G8795 (Sigma-Aldrich, 1:5000), respectively. The species-matched secondary antibodies A5045 (Sigma-Aldrich, 1:5000) and A9044 (Sigma-Aldrich, St. Louis, MO, USA; 1:5000) were used.

### 2.6 X-ray-treatment with increased fractionated doses (growth response assay)

Growth response of cells to X-ray-treatment at dose fractions of ≥ 2 Gy were examined in monolayer cultures. Cells were seeded, monitored, and analyzed as described in section 2.4. After three days (d0) in culture, cells were treated with dose fractions of 2 Gy/d and 5 Gy/d in 24 h intervals for four days. Relative growth (RG) at end of treatment was determined as described elsewhere (33), in short following equation was applied:


$$RG=100\%-100\%*\;\frac{confluence\;{\left[d4\right]}_{treated}-confluence\;{\left[d0\right]}_{treated}}{confluence{\left[d4\right]}_{control}-confluence\;{\left[d0\right]}_{control}}$$

### 2.7 DNA damage response following X-ray-treatment (γH2AX immunostaining)

Cells (4×10^4^/well) were seeded in chamber slides (Ibidi, Gräfelfing, Germany) and grown for 3 days followed by X-ray-treatment with single doses of 0 Gy, 0.5 Gy, or 2 Gy. At 4 and 24 h after irradiation, cells were fixed with 4% (w/v) formaldehyde for 15 min and permeabilized with 0.3% (v/v) Triton X-100 and 0.2% (v/v) Tween20 for 15 min. After blocking with 1% (w/v) bovine serum albumin and 0.2% (v/v) Tween20 for 1 hour, cells were incubated overnight with anti-gammaH2AX phosphoSer139 antibody (#9718S Cell Signaling Technology; Denvers, MA, USA) diluted in blocking buffer (1:400 of 74 µg/mL). Goat anti-rabbit AlexaFluor647 (Invitrogen, ThermoFisher; Waltham, MA USA) was used as secondary antibody diluted in blocking buffer (1:500 of 2 mg/mL). Cell nuclei were stained with Hoechst33258 diluted in Dulbecco’s phosphate-buffered saline (5 µg/mL). Microscopy was performed with the FV12-548–295 confocal laser scanning microscope (Olympus, Tokio, Japan). Quantitative analysis of images (resolution 8.06 px/µm) was performed with ImageJ 1.54f (34) utilizing a threshold-based semi-automated method that identified Hoechst33258-positive nuclei (*“Huang dark no-reset”*, particles size ≥ 25 µm^2^) followed by counting γH2AX-positive foci within each detected nucleus (*“Gaussian Blur”* (sigma = 1), followed by *“Find Maxima”*, prominence = 35). Delineation was checked manually to ensure detection of only whole single cells. Outliers resulting from image artifacts were excluded by ROUT (Q = 1%) as implemented in Prism 10 (GraphPad Software).

### 2.8 Dead cell staining with propidium iodide following X-ray-treatment

Cells (4−6×10^4^/well) were seeded in clear bottom, black wall 96-well microplates (Corning) after 2−3 days, cells were irradiated with a single treatment dose of 5 Gy. 24 hours post irradiation, only designated control dead cells were fixed with 70% (v/v) methanol in DPBS for 30 min at room temperature, followed by incubation of all cells with 4 µg/mL propidium iodide for 40 min at 37°C. Fluorescence intensity was measured at 544 nm (excitation) and 612 nm (emission).

### 2.9 Statistical analysis

Statistical analyses were performed using Prism 10 (GraphPad Software). All data are presented as means ± standard error of the mean. If not stated otherwise, significance of differences was tested using ANOVA analysis with Tukey’s multiple comparison tests.

## 3 Results

### 3.1 X-ray-conditioning to generate radioresistant cell lines by irradiation

Using irradiation with X-rays at sublethal fractionated doses, MPC+EV and MPC+HIF-2α cells were conditioned to acquire a radioresistant phenotype. X-ray-conditioning of the cells using two different irradiation regimes resulted in four MPC sublines, each tolerating fractionated doses up to 2 Gy per day without proliferative arrest (**Table 2**). Both X-ray-conditioning protocols were comparable in terms of time in culture and number of subcultivations, however, the overall time required from parental to endpoint phenotype was shorter with recIR-conditioning since there was no proliferation arrest as with dayIR-conditioning. To harmonize the time endpoints of the different X-ray-conditioning regimes, recIR-conditioning was paused twice (cells were stored in liquid nitrogen) and noIR cultivation was paused once to compensate for the proliferation arrests during dayIR-conditioning.

**Table 2:** Cultivation and dose accumulation during X-ray-conditioning of MPC+EV and MPC+HIF-2α cells.

Cell line	MPC+EV			MPC+HIF-2α		
X-ray-conditioning/maintenance	noIR	dayIR	recIR	noIR	dayIR	recIR
Overall time required [d]	242	242	308	242	242	308
Time in culture [d]	209	242	200	209	242	196
Resting cycles in culture [*n*]	0	3	0	0	3	0
Freeze/thaw cycles [*n*]	1	0	2	1	0	2
Subcultivations [*n*]	34	30	30	34	28	30
Cumulated dose [Gy]	0	201	60	0	201	60

(dayIR) daily irradiation, (recIR) irradiation followed by recovery; (noIR) no irradiation.

X-ray-conditioning of the cells by irradiation and subsequent recovery of their proliferative capacity resulted in the MPC+EV-recIR and MPC+HIF-2α-recIR sublines. Both cell lines accumulated a total radiation dose of 60 Gy. An interruption of this X-ray-conditioning procedure was not necessary as the cells were able to fully recover after each irradiation.

X-ray-conditioning with daily irradiation gave rise to the MPC+EV-dayIR and MPC+HIF-2α-dayIR sublines. Both cell lines accumulated a total radiation dose of 200 Gy. The cells eventually stopped proliferating after initial exposure to fractionated doses of 2 Gy per day. During X-ray-conditioning, MPC+HIF-2α cells showed earlier radiation-induced proliferation arrest compared to MPC+EV cells. Therefore, the irradiation had to be paused three times. Of note, irradiation at low confluence readily caused proliferation arrest. In parallel, cultivation-matched control cells were cultured separately without irradiation, resulting in the MPC+EV-noIR and MPC+HIF-2α-noIR sublines.

### 3.2 Changes in cell morphology, proliferation, and HIF-2α levels during X-ray-conditioning

**Figure 1:**
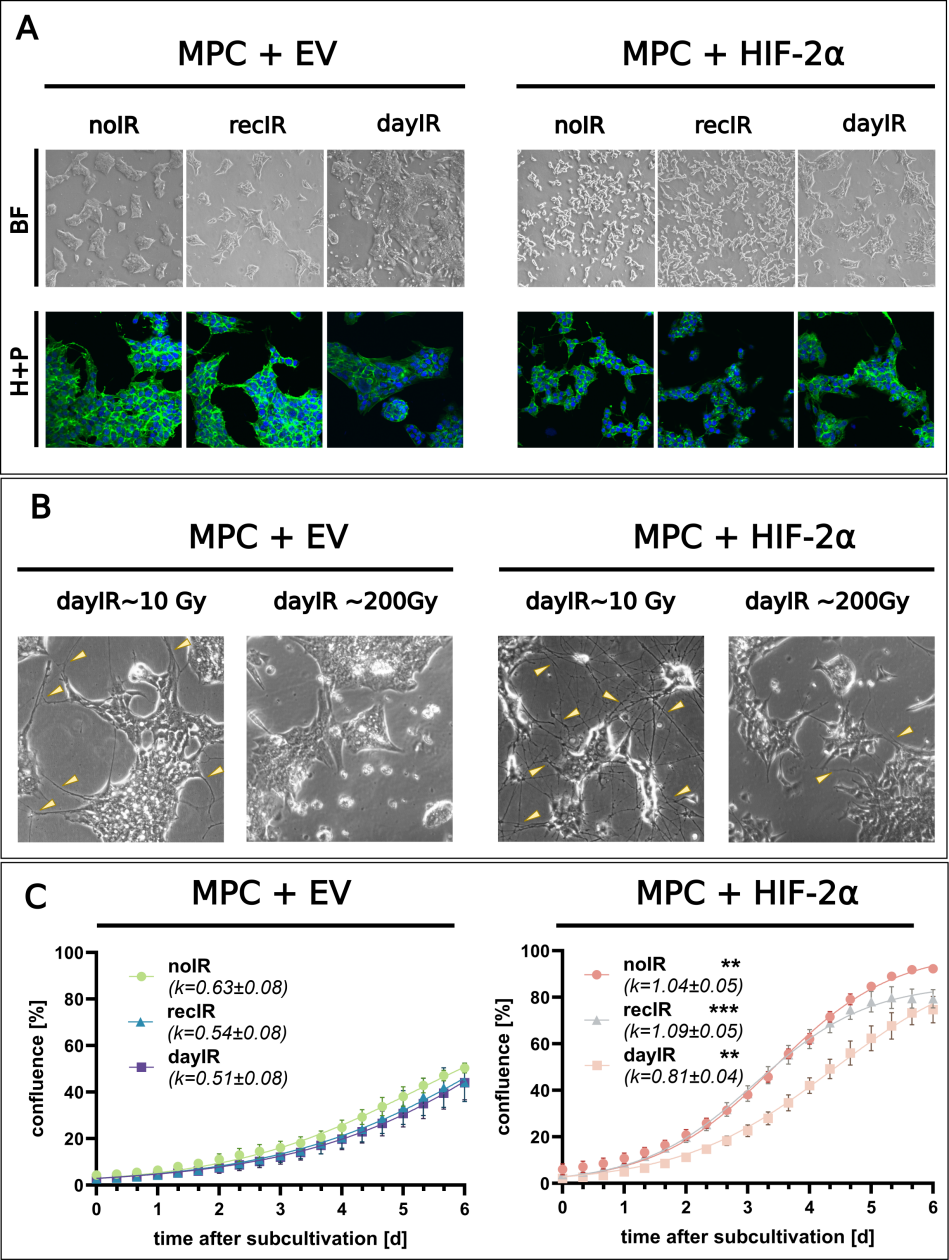
Changes in morphology and proliferation during X-ray-conditioning of MPC+EV and MPC+HIF-2α cells. **(A)** Morphology of X-ray-conditioned cells two weeks after the last irradiation; recIR- and dayIR-conditioned sublines cumulated ~60 and ~200 Gy, respectively; upper panel (BF): bright field images, lower panel (H+P): ICC-IF staining with Hoechst33258 (blue) and Alexa-488 phalloidin (green); dayIR-conditioned cells with increased clustering; **(B)** Enhanced intercellular neurite-like networks (yellow arrows) during dayIR-conditioning at early stages (accumulated dose ~10 Gy) compared to endpoints (accumulated dose ~200 Gy); **(C)** Growth curves based on monolayer confluence two weeks after last irradiation; obtained from 3−5 independent experiments, each including *n* = 10 replicates, growth rates (*k*) determined by curve fitting using the logistic growth equation; significance of growth rate differences compared to corresponding MPC+EV sublines: **p<0.01; ***p<0.001.

X-ray-conditioning had stronger effects on the cellular morphology and proliferation of MPC+HIF-2α cells compared to MPC+EV cells. However, the general cell line-specific differences in morphology, such as the more clustered growth of MPC+EV compared to MPC+HIF-2α cells, were maintained during both recIR- and dayIR-conditioning (**Figures 1A−C**).

Especially when MPC+EV and MPC+HIF-2α cells were subjected to dayIR-conditioning with fractionated doses of ≥1.5 Gy per day, both cell lines responded with an increased density of neurite-like intercellular networks (**Figure 1B**, cumulated dose ~10 Gy). Once the cells had adapted to the daily irradiation, this phenotype regressed. During later stages of dayIR-conditioning with fractionated doses between 1.5 and 2 Gy per day, both MPC+EV and MPC+HIF-2α cells changed toward stronger clustering, even with a tendency toward three-dimensional aggregation (**Figure 1B**, cumulated dose ~200 Gy). In addition, round-structured single cells appeared around the tightly packed cell clusters. This phenotype also reverted when the cells recovered from dayIR-conditioning. These morphological changes also occurred during recIR-conditioning of both MPC+EV and MPC+HIF-2α cells, but were much less pronounced.

DayIR-conditioning had a stronger growth inhibitory effect than recIR-conditioning, especially on MPC+HIF-2α cells. In general, the characteristically faster growth of MPC+HIF-2α cells compared to MPC+EV cells was maintained after both recIR- and dayIR-conditioning, which is also reflected in their growth rates (**Figure 1C**). MPC+HIF-2α-dayIR showed decreased growth compared to MPC+HIF-2α-recIR and MPC+HIF-2α-noIR, but proliferated faster than all MPC+EV cell lines. It is noteworthy that all the MPC+EV cell lines never reached 100% confluence due to extensive clustering. The growth rates of MPC+EV cells remained largely similar with only slight decreases in the corresponding X-ray-conditioned sublines.

**Figure 2:**
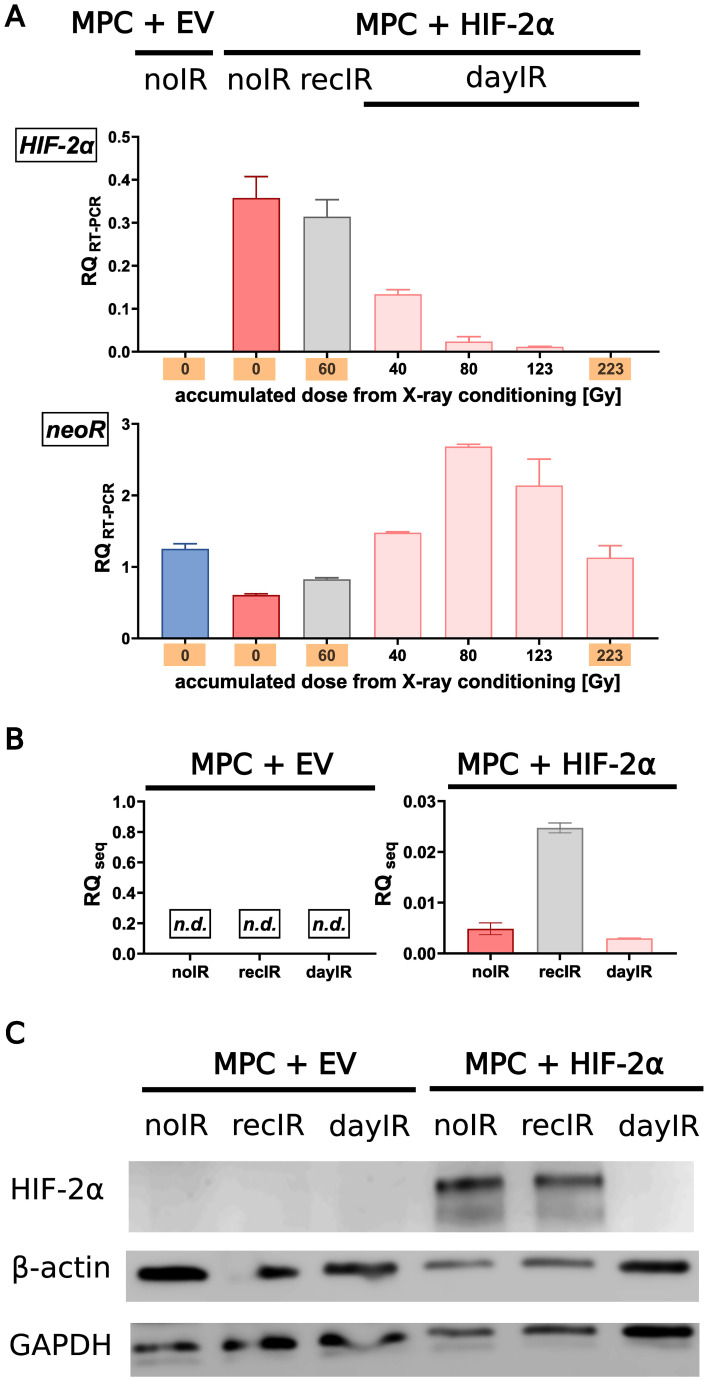
Gradual loss of HIF-2α during dayIR-conditioning of genetically modified MPC+HIF-2α cells. While both unconditioned (noIR) and recIR-conditioned MPC+HIF-2α cells retained their cell line-specific HIF-2α-positive phenotype, dayIR-conditioning led to a gradual reduction in *HIF-2α* transgene expression in a dose-dependent manner that ultimately resulted in loss of HIF-2α; **(A)** Expression of the *HIF-2α* and *neoR* transgenes over the course of X-ray-conditioning at increasing intermediate and final accumulated doses (orange); relative quantity of RT-PCR products (RQ_RT-PCR_, normalized to murine *Actb)* amplified from RNA isolates, *n* = 3; **(B)** Residual expression of the *HIF-2α* transgene detected by RNA sequencing of samples collected at the endpoints of X-ray-conditioning; relative quantity of transcripts (RQ_seq_, normalized to murine *Actb*), *n* = 3; **(C)** HIF-2α protein levels at the endpoints of X-ray-conditioning; lysates prepared from cells exposed to CoCl_2_ (HIF stabilizer) for 24 h, band intensities compared to β-actin and glyceraldehyde phosphate dehydrogenase (GAPDH).

Molecular analyses of the cell samples showed that the MPC+HIF-2α cell lines retained their cell line-specific HIF-2α-positive phenotype during recIR-conditioning, whereas dayIR-conditioning resulted in a dose-dependent reduction in the *HIF-2α* transgene expression (**Figures 2A, B**) and ultimate loss of HIF-2α (**Figure 2C**). This most likely explains the reduced growth rate of this cell line. Of note, both MPC+HIF-2α-recIR and MPC+HIF-2α-dayIR cells retained expression of the *neoR* transgene, which is located on the episomal *HIF-2α* expression vector and confers resistance against G418.

### 3.3 Resistance to X-ray-treatment at increasing fractionated doses

**Figure 3:**
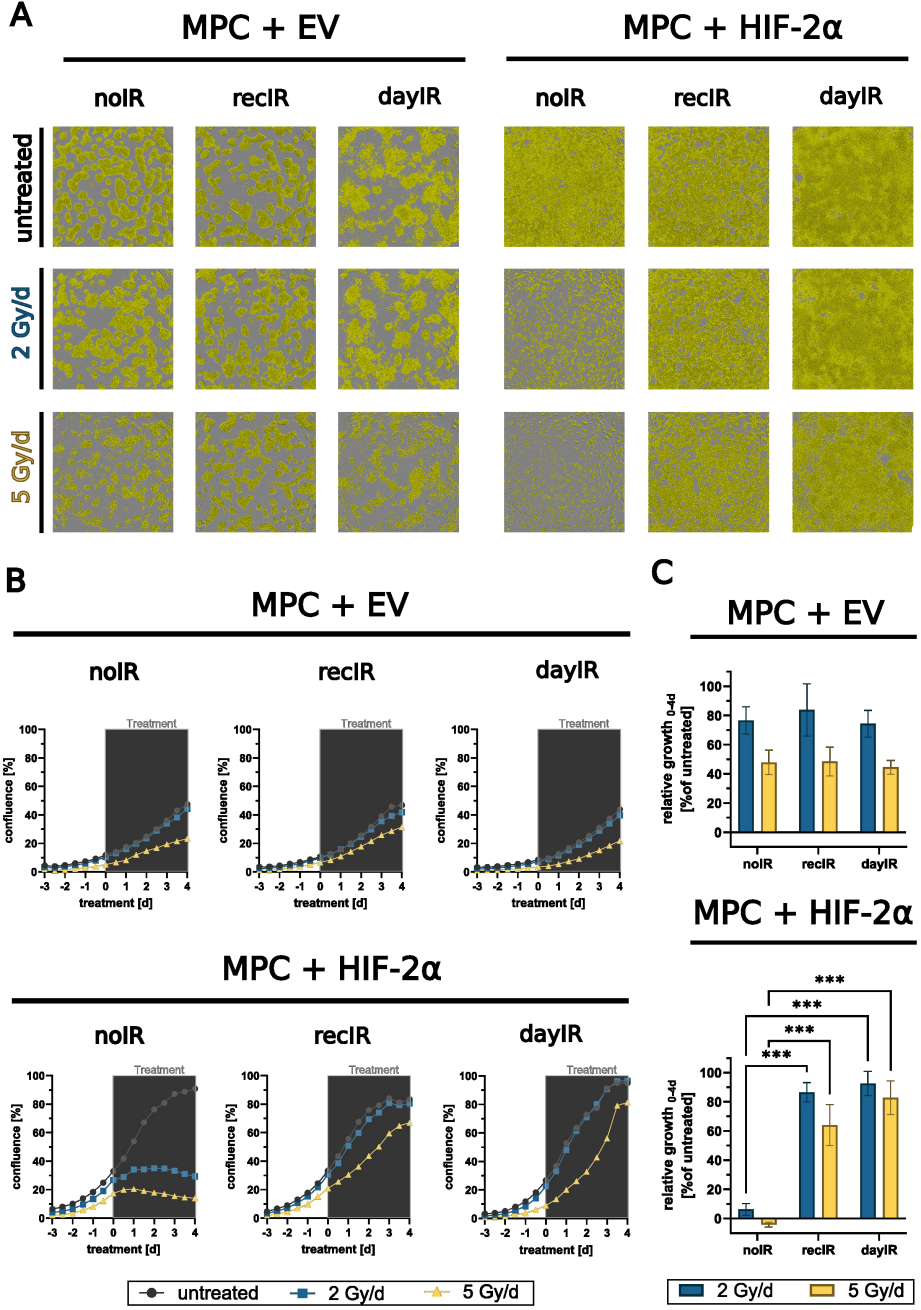
X-ray-conditioning enabled sustained proliferation of MPC+HIF-2α cells during X-ray-treatment. Growth responses in monolayers during X-ray-treatment with increasing fractionated doses over four days; MPC+EV-noIR, -recIR, and -dayIR cell lines all showed similar growth responses; unconditioned MPC+HIF-2α-noIR cells stopped proliferating during X-ray-treatment, while both X-ray-conditioned MPC+HIF-2α-recIR and -dayIR sublines maintained proliferation; **(A)** Microscopic images with confluence masks (yellow) at the end of X-ray-treatment (day 4); **(B)** Changes in confluence over time; means of one experiment including *n* = 10 replicates; (gray box) duration of X-ray-treatment with fractionated doses in 24-hour intervals; **(C)** Relative growth at the end of X-ray-treatment (day 4) normalized to the corresponding untreated controls and to the respective confluence at treatment start (day 0), means of three independent experiments each including *n* = 10 replicates, significance of differences compared to unconditioned noIR controls: ***p<0.001; all experiments were conducted two weeks after last X-ray-conditioning.

After two weeks of recovery from X-ray-conditioning, MPC+EV-recIR and -dayIR cells as well as MPC+HIF-2α-recIR and -day IR cells still exhibited characteristics of increased radioresistance to X-ray-treatment for 4 days with fractionated doses of 2 and 5 Gy per day (**Figures 3A−C**). MPC+EV cells retained their already higher ability to sustain proliferation. Even without X-ray-conditioning, MPC+EV-noIR cells maintained 80% and 40% of their normal growth during X-ray-treatment with 2 and 5 Gy per day, respectively. Likewise, both X-ray-conditioned MPC+EV-recIR and -dayIR sublines showed the same dose responses. MPC without X-ray-conditioning (MPC+HIF-2α-noIR) came to a complete arrest during X-ray-treatment with 2 and 5 Gy per day. In contrast, the X-ray-conditioned MPC+HIF-2α-recIR- and -dayIR sublines maintained 83% and 64% of their normal growth, respectively, even during X-ray-treatment with fractionated doses of 5 Gy per day.

**Figure 4:**
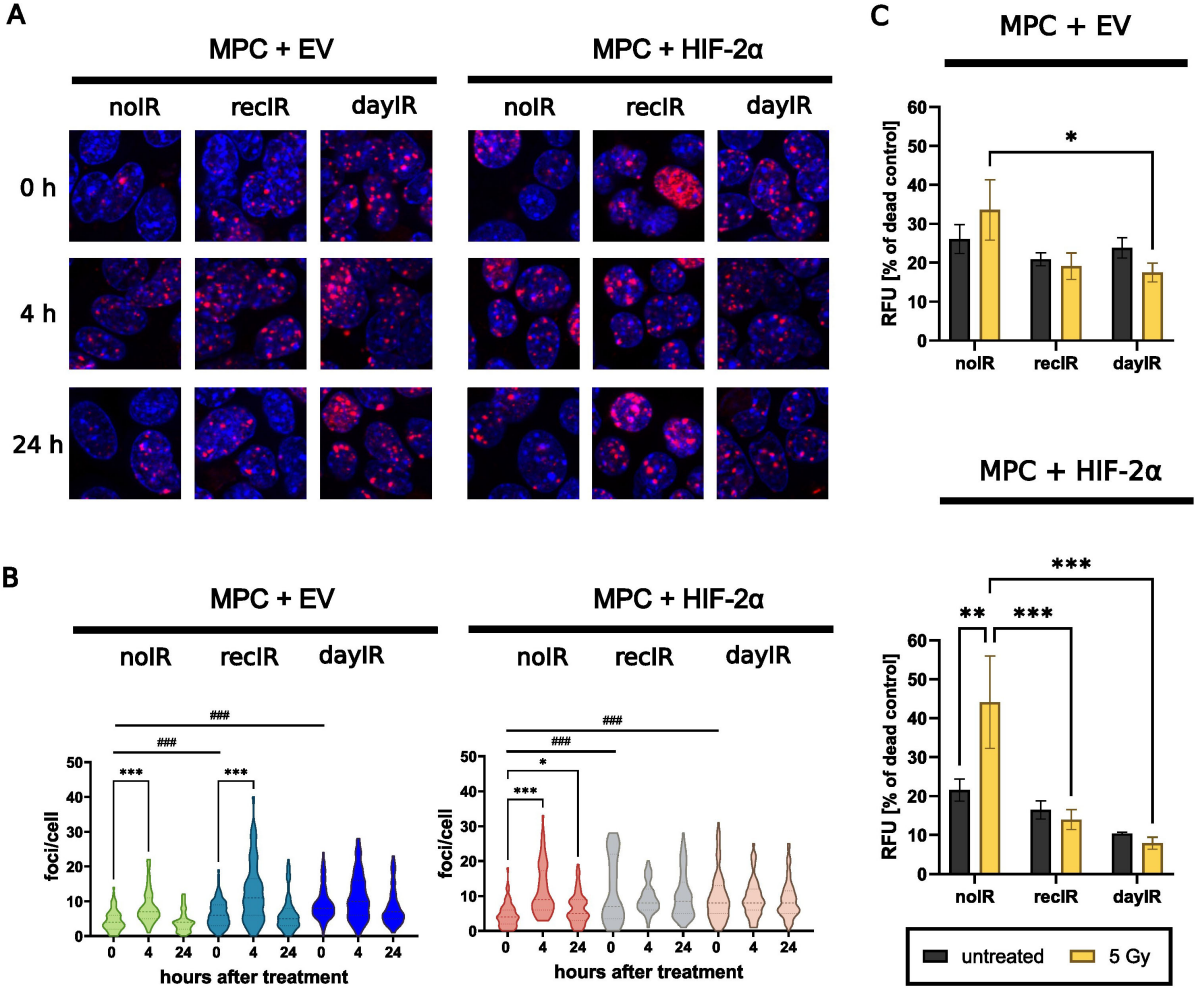
X-ray-conditioning increases DNA double strand break repair and reduces proportions of dead MPC+EV and MPC+HIF-2α cells after X-ray-treatment. **(A)** Immunostaining of γH2AX foci (red) and nuclei counterstained with Hoechst 33258 (blue) after X-ray-treatment of cells with 2 Gy; **(B)** Quantitative image analysis of γH2AX foci per cell showing that irradiation induced DNA repair in both unconditioned MPC+EV and MPC+HIF-2α cells, while all recIR- and dayIR-conditioned sublines already showed increased prevalence of DNA repair; statistical distribution and means (dashed lines) from >20 nuclei; significance of differences compared to the respective noIR cell lines: ^###^ *p*<0.001 and compared to the same X-ray-conditioned cell line without acute irradiation: *p<0.05; ***p<0.001; **(C)** Proportions of dead cells after X-ray-treatment with 5 Gy; cells stained with propidium iodide 24 h after irradiation; relative fluorescence intensity of dead cells normalized to the maximum amount of dead cells determined from exposure to methanol; two independent experiments each including *n* = 9 replicates; significance of differences: *p<0.05; **°*p*<0.01; ***p<0.001; all experiments were conducted two weeks after last X-ray-conditioning.

X-ray-conditioned MPC+EV and MPC+HIF-2α cells showed increased capacity for DNA repair, as evidenced by an increased number of γH2AX foci per cell (**Figures 4A, B**). Even without X-ray-treatment (0 hours), the initial number of γH2AX foci was already significantly higher in all four recIR- and dayIR-conditioned MPC+EV and MPC+HIF-2α cell sublines compared to their corresponding unconditioned noIR controls. Unconditioned MPC+EV-noIR and MPC+HIF-2α-noIR cells responded to X-ray-treatment with a single dose of 2 Gy with a transient increase in γH2AX foci reaching a maximum after 4 hours, followed by a decrease to initial levels within 24 hours. In both X-ray-conditioned MPC+EV-recIR and -dayIR cells, X-ray-treatment led to an additional increase in pre-elevated γH2AX foci and showed a similar time dependence as in unconditioned MPC+EV-noIR cells. In contrast, X-ray-conditioned MPC+HIF-2α-recIR and -dayIR cells maintained their pre-elevated γH2AX foci largely unchanged 4 and 24 hours after X-ray-treatment.

X-ray-conditioned MPC+EV and MPC+HIF-2α cells showed reduced proportions of dead cells detectable in monolayer cultures 24 hours after X-ray-treatment (**Figure 4C**). A single X-ray-treatment dose of 5 Gy increased the proportions of dead unconditioned MPC+EV-noIR cells (1.3-fold) and MPC+HIF-2α-noIR cells (2.1-fold), while the proportions of dead recIR- and dayIR-conditioned MPC+EV and MPC+HIF-2α cells remained largely unchanged.

## 4 Discussion

We have succeeded in establishing four MPC cell lines that exhibit an X-ray-induced phenotype with increased radioresistance, with the largest adaptations occurring in HIF-2α-positive MPC sublines. The starting point for this methodological approach was the MPC cell line derived from an adrenal pheochromocytoma of a neurofibromin-1-knockout mouse, which has already been extensively characterized and is considered an established model for basic and preclinical investigations on this tumor entity (20, 35–37).

For both induction and subsequent testing of radioresistance in MPC+EV and MPC+HIF-2α cells, we preferred external irradiation with X-rays over treatment with particle-emitting radioligands. External irradiation assured that both cell lines received the same radiation dose throughout the study, thus avoiding potential dose inaccuracies resulting from different concentrations of molecular targets involved in radioligand binding. For example, previous studies have shown that HIF-2α positivity is associated with lower uptake of SSTR2 radioligands in MPC cells (24).

For inducing radioresistance in MPC cells, we applied two X-ray-conditioning regimes utilizing irradiation of cellular monolayers with sublethal fractionated doses of up to 2 Gy per irradiation: either daily (dayIR) or with full recovery in confluence between each irradiation step (recIR). X-ray-conditioning of MPC+EV and MPC+HIF-2α cells following the recIR regime was less elaborate and easier to manage compared to the dayIR regime. Due to full proliferative recovery of the cells between each X-ray-conditioning step, the irradiation schedule for recIR-conditioning could be easily harmonized with the seeding density of cells during subcultivation. The dayIR-conditioning regime was more demanding and time-consuming since daily irradiations had to be conducted with only little flexibility in schedule, and cells, especially the MPC+HIF-2α sublines, frequently stopped proliferating under fractionated doses of ≥1.5 Gy per day.

X-ray-conditioning with both recIR and dayIR regimes was associated with changes in morphology and growth of MPC+EV and MPC+HIF-2α cells. Cells increased their density in neurite-like networks when fractionated doses ≥ 1.5 Gy per day were applied for the first time. After accumulating a total dose of approximately 10 Gy, delivered in fractionated doses of 1.5 or 2 Gy per day, this extensive phenotype receded, marking the first major adaptation to irradiation-induced cellular stress. DayIR-conditioning of MPC cells was associated with a shift in morphology toward increased clustering as well as a decrease in growth rates. The reason why these effects occurred specifically after dayIR-conditioning might be connected to the higher accumulated doses of ~200 Gy in dayIR-conditioned cells *versus* ~60 Gy in recIR-conditioned cells. A decrease in growth rates has also been reported previously for radioresistant prostate cancer cell lines derived from surviving clones of irradiated tumors (38) and for radioresistant breast cancer cell lines generated through X-ray-conditioning, where the reduction in proliferation was linked to a reduced expression of the *MKI67* gene encoding the marker of proliferation Ki67 (19). Furthermore, decreased proliferation of chemoresistant carcinoma cell lines of different origins has been reported to avoid cytotoxic actions of cancer drugs (39). In line with this, our results indicate that the dayIR-conditioned MPC cells may use a similar cytostatic mechanism of delaying growth to enable efficient DNA repair and to avoid accumulation of potentially lethal DNA double strand breaks.

During dayIR-conditioning with fractionated doses of 1.5 and 2 Gy per day, MPC+EV cells were still proliferating, whereas MPC+HIF-2α frequently stopped proliferation. The same cell line-specific responses occurred after X-ray-treatment of unconditioned MPC+EV-noIR and MPC+HIF-2α-noIR cells with fractionated doses of 2 and 5 Gy per day. This indicates that MPC+EV cells are generally more robust to X-ray irradiation than MPC+HIF-2α cells. These cell line-specific sensitivity to irradiation might be related to the different biology of MPC+EV and MPC+HIF-2α cells, namely faster growth of MPC+HIF-2α cells and stronger clustering of MPC+EV cells. DayIR-conditioning of MPC+HIF-2α was associated with increased clustering and slower growth, suggesting that intercellular connectivity and harmonized proliferation arrest may confer resistance to irradiation in these cells. Furthermore, the weaker morphologic and proliferative responses of MPC+EV cells to irradiation compared to MPC+HIF-2α cells point toward an inherent radioresistance of MPC+EV cells, which may primarily be determined by their connective clustering and not by lack of HIF-2α signaling. There is growing evidence, mostly from studies in brain tumors, that by formation of interconnected cellular networks, cancer cells can collectively sense and respond to therapeutic attacks, share resources or protective signals, and repair molecular damage, thereby enhancing their survival and acquire therapy resistance (40). However, the contributions of HIF-2α to radioresistance would become more apparent in more advanced culture models, such as three-dimensional tumor spheroids and allografts. Our previous work already showed increased radioresistance of HIF-2α-positive MPC tumor spheroids over those lacking HIF-2α (20).

Round-structured cells that occurred during X-ray-conditioning are most likely senescent cells. Senescence is triggered by stressful insults and results in senescent cells that stop multiplying but on the other hand do not die off (41). The observation, that round-structured cells occurred predominantly during dayIR-conditioning of both MPC+EV and MPC+HIF-2α cells, but disappeared during recovery, suggests a temporary increase in senescent cells in response to X-ray-conditioning.

During dayIR-conditioning of genetically modified MPC + HIF-2α cells, a dose-dependent reduction in the *HIF-2α* transgene expression occurred, resulting in ultimate loss of HIF-2α. This loss of HIF-2α was associated with morphologic changes toward more clustered and slower growth of the cells suggesting that discontinued HIF-2α signaling is involved in this phenotype reversal. This is also supported by previous reports demonstrating that a clustered growth is more pronounced in HIF-2α-negative compared to HIF-2α-positive MPC cells (35). Since dayIR-conditioning with daily doses of ≥ 1.5 Gy was largely intolerable for MPC+HIF-2α cells, we hypothesize, that the amount of DNA damage may have led to cell clones with a damaged *HIF-2α* expression cassette resulting in non-functional or unstable transcripts. Since recIR-conditioning of MPC+HIF-2α-cells resulted in increased radioresistance while still preserving the HIF-2α-positive phenotype, MPC+HIF-2α-recIR will be the preferred model to investigate the possible role of HIF-2α in modulating radioresistance in PCC/PGL.

Two weeks after successful X-ray-conditioning, X-ray-treatment at higher doses between 2 and 5 Gy confirmed that MPC+EV-recIR and -dayIR cells as well as MPC+HIF-2α-recIR and -dayIR cells maintain characteristics of an X-ray-induced radioresistant phenotype, based on their cellular responses in proliferation, DNA double strand break repair, and cell death. In this context, we successfully evaluated the cytotoxic effects of X-ray-treatment on proliferation of MPC cells using both image-based quantification of monolayer confluence and staining of dead cells with propidium iodide. Both methods represent alternatives to the measurement of ‘clonogenic’ growth, which is one of the most commonly used assay formats to test cytotoxic effects on cells *in vitro* (42). The clonogenic assay methodology essentially requires the analysis of colony numbers re-grown from a homogeneous single-cell suspension. However, MPC cells are difficult to study in clonogenic assays because they have strong tendency to form aggregates and cannot be reliably grown from single cell seeding. For this reason, we used experimental setups that tolerate higher cell densities instead of single-cell seeding and have already been successfully used to evaluate radiosensitivity *in vitro* (43).

X-ray-treatment of unconditioned MPC+EV-noIR and MPC+HIF-2α-noIR cells with doses increasing from 2 to 5 Gy per day was associated with a decrease of the maximal confluence. This is consistent with the higher proportions of dead cells detected in propidium iodide stain after X-ray-treatment with 5 Gy. At the same dose, the decrease in confluence of X-ray-conditioned MPC+EV-dayIR and -recIR cells remained unchanged, while the proportions of dead cells decreased compared to unconditioned MPC+EV-noIR, indicating that these cells resist irradiation in a state of proliferative arrest without induction of cell death mechanisms. The responses of X-ray-conditioned MPC+HIF-2α-dayIR and -recIR cells to X-ray-treatments are characterized by both sustaining proliferation and escaping cell death induction.

X-ray-conditioning of both MPC+EV and MPC+HIF-2α cells was associated with elevated γH2AX foci. Furthermore, γH2AX foci were temporary induced after acute X-ray treatment in unconditioned MPC+EV-noIR and MPC+HIF-2α-noIR-cells but remained continuously high in the corresponding recIR- and dayIR-conditioned sublines. These results demonstrate that a temporary induction of DNA double strand break repair after X-ray-treatment is characteristic for the more radiosensitive (noIR) MPC cell lines, while the more radioresistant cell lines (dayIR- and recIR-conditioned) retain constantly higher capacity in DNA double strand break repair. Pre-elevated γH2AX foci indicate that kinases for phosphorylation of H2AX are constantly present and activated in large numbers. For example, the phosphorylation of H2AX is primarily mediated by members of the PI3-kinase-related kinase family, particularly ataxia telangiectasia mutated (ATM), DNA-dependent protein kinase catalytic subunit, and ataxia telangiectasia mutated and Rad3-related (ATR) (44–46). Additionally, other mechanisms may also have contributed to higher γH2AX foci in X-ray-conditioned MPC cell lines, such as irradiation-induced telomere shortening and higher numbers of senescent and apoptotic cells (47–49).

## 5 Conclusion

The present study introduces a series of X-ray-conditioned MPC+EV and MPC+HIF-2α cell lines with upregulated DNA repair capacity that maintain proliferation under sustained exposure to fractionated X-ray doses of up to 5 Gy per day. These cell lines are therefore considered suitable models to study morphological, physiological, and transcriptional alterations associated with radioresistance in PCC/PGL, including the potential role of HIF-2α in evading radiotherapeutic control and inducing metastatic eruption. Given the inherent characteristics of the MPC+EV and MPC+HIF-2α cell lines, their X-ray-conditioned variants are likely suitable for further investigations not only in monolayer cultures but also in more advanced three-dimensional models such a tumor spheroids and tumor allografts in mice.

## Data Availability

The original contributions presented in the study are included in the article/supplementary material. Further inquiries can be directed to the corresponding authors.

## References

[1] Fishbein L Leshchiner I Walter V Danilova L Robertson AG Johnson AR . Comprehensive molecular characterization of pheochromocytoma and paraganglioma. Cancer Cell. (2017) 31:181–93. doi: 10.1016/j.ccell.2017.01.001, PMID: 28162975 PMC5643159

[2] Crona J Lamarca A Ghosal S Welin S Skogseid B Pacak K . Genotype–phenotype correlations in pheochromocytoma and paraganglioma: a systematic review and individual patient meta-analysis(2019). Available online at: https://erc.bioscientifica.com/view/journals/erc/26/5/ERC-19-0024.xml (Accessed June 10, 2025)., PMID: 10.1530/ERC-19-0024 PMC6717695 30893643

[3] Huang BL Liu Q Teng YY Peng SQ Liu Z Li ML . Global trends and current status in pheochromocytoma: a bibliometric analysis of publications in the last 20 years. Front Endocrinol. (2023) 14:1167796. doi: 10.3389/fendo.2023.1167796, PMID: 37680890 PMC10482340

[4] Jain A Baracco R Kapur G . Pheochromocytoma and paraganglioma-an update on diagnosis, evaluation, and management. Pediatr Nephrol Berl Ger. (2020) 35:581–94. doi: 10.1007/s00467-018-4181-2, PMID: 30603807

[5] Conzo G Pasquali D Colantuoni V Circelli L Tartaglia E Gambardella C . Current concepts of pheochromocytoma. Int J Surg Lond Engl. (2014) 12:469–74. doi: 10.1016/j.ijsu.2014.04.001, PMID: 24727002

[6] Lam AKY . Update on adrenal tumours in 2017 world health organization (WHO) of endocrine tumours. Endocr Pathol. (2017) 28:213–27. doi: 10.1007/s12022-017-9484-5, PMID: 28477311

[7] Salmenkivi K Heikkilä P Haglund C Arola J . Malignancy in pheochromocytomas. APMIS Acta Pathol Microbiol Immunol Scand. (2004) 112:551–9. doi: 10.1111/j.1600-0463.2004.apm1120901.x, PMID: 15601303

[8] Yu L Fleckman AM Chadha M Sacks E Levetan C Vikram B . Radiation therapy of metastatic pheochromocytoma: case report and review of the literature. Am J Clin Oncol. (1996) 19:389–93. doi: 10.1097/00000421-199608000-00015, PMID: 8677912

[9] Wolf KI Jha A van Berkel A Wild D Janssen I Millo CM . Eruption of metastatic paraganglioma after successful therapy with 177Lu/90Y-DOTATOC and 177Lu-DOTATATE. Nucl Med Mol Imaging. (2019) 53:223–30. doi: 10.1007/s13139-019-00579-w, PMID: 31231443 PMC6554376

[10] Keith B Johnson RS Simon MC . HIF1α and HIF2α: sibling rivalry in hypoxic tumour growth and progression. Nat Rev Cancer. (2011) 12:9–22. doi: 10.1038/nrc3183, PMID: 22169972 PMC3401912

[11] Zhao J Du F Luo Y Shen G Zheng F Xu B . The emerging role of hypoxia-inducible factor-2 involved in chemo/radioresistance in solid tumors. Cancer Treat Rev. (2015) 41:623–33. doi: 10.1016/j.ctrv.2015.05.004, PMID: 25981453

[12] Richter S Steenblock C Fischer A Lemm S Ziegler CG Bechmann N . Improving susceptibility of neuroendocrine tumors to radionuclide therapies: personalized approaches towards complementary treatments. Theranostics. (2024) 14:17–32. doi: 10.7150/thno.87345, PMID: 38164150 PMC10750207

[13] Eisenhofer G Huynh TT Pacak K Brouwers FM Walther MM Linehan WM . Distinct gene expression profiles in norepinephrine- and epinephrine-producing hereditary and sporadic pheochromocytomas: activation of hypoxia-driven angiogenic pathways in von Hippel–Lindau syndrome(2004). Available online at: https://erc.bioscientifica.com/view/journals/erc/11/4/0110897.xml (Accessed June 10, 2025)., PMID: 10.1677/erc.1.00838 15613462

[14] Burnichon N Vescovo L Amar L Libé R de Reynies A Venisse A . Integrative genomic analysis reveals somatic mutations in pheochromocytoma and paraganglioma. Hum Mol Genet. (2011) 20:3974–85. doi: 10.1093/hmg/ddr324, PMID: 21784903

[15] López-Jiménez E Gómez-López G Leandro-García LJ Muñoz I Schiavi F Montero-Conde C . Research resource: transcriptional profiling reveals different pseudohypoxic signatures in SDHB and VHL-related pheochromocytomas. Mol Endocrinol. (2010) 24:2382–91. doi: 10.1210/me.2010-0256, PMID: 20980436 PMC5417372

[16] Favier J Brière JJ Burnichon N Rivière J Vescovo L Benit P . The warburg effect is genetically determined in inherited pheochromocytomas. PloS One. (2009) 4:e7094. doi: 10.1371/journal.pone.0007094, PMID: 19763184 PMC2738974

[17] Lohberger B Glänzer D Eck N Kerschbaum-Gruber S Mara E Deycmar S . Activation of efficient DNA repair mechanisms after photon and proton irradiation of human chondrosarcoma cells. Sci Rep. (2021) 11:24116. doi: 10.1038/s41598-021-03529-9, PMID: 34916568 PMC8677811

[18] Kuwahara Y Roudkenar MH Urushihara Y Saito Y Tomita K Roushandeh AM . Clinically relevant radioresistant cell line: a simple model to understand cancer radioresistance. Med Mol Morphol. (2017) 50:195–204. doi: 10.1007/s00795-017-0171-x, PMID: 29067564

[19] Gray M Turnbull AK Ward C Meehan J Martínez-Pérez C Bonello M . Development and characterisation of acquired radioresistant breast cancer cell lines. Radiat Oncol. (2019) 14:64. doi: 10.1186/s13014-019-1268-2, PMID: 30987655 PMC6466735

[20] Seifert V Richter S Bechmann N Bachmann M Ziegler CG Pietzsch J . HIF2alpha-associated pseudohypoxia promotes radioresistance in pheochromocytoma: insights from 3D models. Cancers. (2021) 13:385. doi: 10.3390/cancers13030385, PMID: 33494435 PMC7865577

[21] Roig EM Groot AJ Yaromina A Hendrickx TC Barbeau LMO Giuranno L . HIF-1α and HIF-2α Differently regulate the radiation sensitivity of NSCLC cells. Cells. (2019) 8:45. doi: 10.3390/cells8010045, PMID: 30642030 PMC6356534

[22] Fukuda K Sakakura C Miyagawa K Kuriu Y Kin S Nakase Y . Differential gene expression profiles of radioresistant oesophageal cancer cell lines established by continuous fractionated irradiation. Br J Cancer. (2004) 91:1543–50. doi: 10.1038/sj.bjc.6602187, PMID: 15365572 PMC2409931

[23] Ullrich M Bergmann R Peitzsch M Cartellieri M Qin N Ehrhart-Bornstein M . *In vivo* fluorescence imaging and urinary monoamines as surrogate biomarkers of disease progression in a mouse model of pheochromocytoma. Endocrinology. (2014) 155:4149–56. doi: 10.1210/en.2014-1431, PMID: 25137029 PMC4256828

[24] Seifert V Liers J Kniess T Richter S Bechmann N Feldmann A . Fluorescent mouse pheochromocytoma spheroids expressing hypoxia-inducible factor 2 alpha: Morphologic and radiopharmacologic characterization. J Cell Biotechnol. (2019) 5:135–51. doi: 10.3233/JCB-199005

[25] Qin N de Cubas AA Garcia-Martin R Richter S Peitzsch M Menschikowski M . Opposing effects of HIF1α and HIF2α on chromaffin cell phenotypic features and tumor cell proliferation: Insights from MYC-associated factor X. Int J Cancer. (2014) 135:2054–64. doi: 10.1002/ijc.28868, PMID: 24676840

[26] Babraham Bioinformatics . FastQC A Quality Control tool for High Throughput Sequence Data. Available online at: https://www.bioinformatics.babraham.ac.uk/projects/fastqc/ (Accessed May 7, 2025).

[27] Bolger AM Lohse M Usadel B . Trimmomatic: a flexible trimmer for Illumina sequence data. Bioinforma Oxf Engl. (2014) 30:2114–20. doi: 10.1093/bioinformatics/btu170, PMID: 24695404 PMC4103590

[28] Dobin A Davis CA Schlesinger F Drenkow J Zaleski C Jha S . STAR: ultrafast universal RNA-seq aligner. Bioinforma Oxf Engl. (2013) 29:15–21. doi: 10.1093/bioinformatics/bts635, PMID: 23104886 PMC3530905

[29] Danecek P Bonfield JK Liddle J Marshall J Ohan V Pollard MO . Twelve years of SAMtools and BCFtools. GigaScience. (2021) 10:giab008. doi: 10.1093/gigascience/giab008, PMID: 33590861 PMC7931819

[30] Liao Y Smyth GK Shi W . featureCounts: an efficient general purpose program for assigning sequence reads to genomic features. Bioinforma Oxf Engl. (2014) 30:923–30. doi: 10.1093/bioinformatics/btt656, PMID: 24227677

[31] Love MI Huber W Anders S . Moderated estimation of fold change and dispersion for RNA-seq data with DESeq2. Genome Biol. (2014) 15:550. doi: 10.1186/s13059-014-0550-8, PMID: 25516281 PMC4302049

[32] Ewels P Magnusson M Lundin S Käller M . MultiQC: summarize analysis results for multiple tools and samples in a single report. Bioinforma Oxf Engl. (2016) 32:3047–8. doi: 10.1093/bioinformatics/btw354, PMID: 27312411 PMC5039924

[33] Ullrich M Bergmann R Peitzsch M Zenker EF Cartellieri M Bachmann M . Multimodal somatostatin receptor theranostics using [64Cu]Cu-/[177Lu]Lu-DOTA-(Tyr3)octreotate and AN-238 in a mouse pheochromocytoma model. Theranostics. (2016) 6:650–65. doi: 10.7150/thno.14479, PMID: 27022413 PMC4805660

[34] Schneider CA Rasband WS Eliceiri KW . NIH Image to ImageJ: 25 years of image analysis. Nat Methods. (2012) 9:671–5. doi: 10.1038/nmeth.2089, PMID: 22930834 PMC5554542

[35] Bechmann N Moskopp ML Ullrich M Calsina B Wallace PW Richter S . HIF2α supports pro-metastatic behavior in pheochromocytomas/paragangliomas. Endocr Relat Cancer. (2020) 27:625–40. doi: 10.1530/ERC-20-0205, PMID: 33112842

[36] Powers JF Evinger MJ Tsokas P Bedri S Alroy J Shahsavari M . Pheochromocytoma cell lines from heterozygous neurofibromatosis knockout mice. Cell Tissue Res. (2000) 302:309–20. doi: 10.1007/s004410000290, PMID: 11151443

[37] Wang K Fischer A Maccio U Hantel C Beuschlein F Grossman AB . Pre-clinical phaeochromocytoma and paraganglioma models: Cell lines, animal models, and a human primary culture model. Best Pract Res Clin Endocrinol Metab. (2024) 38(6):101913. doi: 10.1016/j.beem.2024.101913, PMID: 38972796

[38] Chang L Ni J Beretov J Wasinger VC Hao J Bucci J . Identification of protein biomarkers and signaling pathways associated with prostate cancer radioresistance using label-free LC-MS/MS proteomic approach. Sci Rep. (2017) 7:41834. doi: 10.1038/srep41834, PMID: 28225015 PMC5320484

[39] Sazonova EV Yapryntseva MA Pervushin NV Tsvetcov RI Zhivotovsky B Kopeina GS . Cancer drug resistance: targeting proliferation or programmed cell death. Cells. (2024) 13:388. doi: 10.3390/cells13050388, PMID: 38474352 PMC10930385

[40] Schneider M Potthoff AL Karpel-Massler G Schuss P Siegelin MD Debatin KM . The Alcatraz-Strategy: a roadmap to break the connectivity barrier in Malignant brain tumours. Mol Oncol. (2024) 18:2890–905. doi: 10.1002/1878-0261.13642, PMID: 38567664 PMC11619800

[41] Gorgoulis V Adams PD Alimonti A Bennett DC Bischof O Bishop C . Cellular senescence: defining a path forward. Cell. (2019) 179:813–27. doi: 10.1016/j.cell.2019.10.005, PMID: 31675495

[42] Brix N Samaga D Belka C Zitzelsberger H Lauber K . Analysis of clonogenic growth *in vitro* . Nat Protoc. (2021) 16:4963–91. doi: 10.1038/s41596-021-00615-0, PMID: 34697469

[43] Kuwahara Y Mori M Oikawa T Shimura T Ohtake Y Mori S . The modified high-density survival assay is the useful tool to predict the effectiveness of fractionated radiation exposure. J Radiat Res (Tokyo). (2010) 51:297–302. doi: 10.1269/jrr.09094, PMID: 20410675

[44] Burma S Chen BP Murphy M Kurimasa A Chen DJ . ATM phosphorylates histone H2AX in response to DNA double-strand breaks. J Biol Chem. (2001) 276:42462–7. doi: 10.1074/jbc.C100466200, PMID: 11571274

[45] Stiff T O’Driscoll M Rief N Iwabuchi K Löbrich M Jeggo PA . ATM and DNA-PK function redundantly to phosphorylate H2AX after exposure to ionizing radiation. Cancer Res. (2004) 64:2390–6. doi: 10.1158/0008-5472.CAN-03-3207, PMID: 15059890

[46] Hanasoge S Ljungman M . H2AX phosphorylation after UV irradiation is triggered by DNA repair intermediates and is mediated by the ATR kinase. Carcinogenesis. (2007) 28:2298–304. doi: 10.1093/carcin/bgm157, PMID: 17615256

[47] Sishc BJ Nelson CB McKenna MJ Battaglia CLR Herndon A Idate R . Telomeres and telomerase in the radiation response: implications for instability, reprograming, and carcinogenesis. Front Oncol. (2015) 5:257. doi: 10.3389/fonc.2015.00257, PMID: 26636039 PMC4656829

[48] Olive PL . Endogenous DNA breaks: γH2AX and the role of telomeres. Aging. (2009) 1:154–6. doi: 10.18632/aging.100025, PMID: 20157507 PMC2806006

[49] Bernadotte A Mikhelson VM Spivak IM . Markers of cellular senescence. Telomere shortening as a marker of cellular senescence. Aging. (2016) 8:3–11. doi: 10.18632/aging.100871, PMID: 26805432 PMC4761709

